# Unraveling behavioral and sociocultural factors that shape antimicrobial use among patients and general public, Addis Ababa, Ethiopia, a qualitative study

**DOI:** 10.1186/s40780-025-00503-9

**Published:** 2025-10-29

**Authors:** Oumer Sada Muhammed, Mirgissa Kaba Serbessa, Teferi Gedif Fenta

**Affiliations:** 1 Department of Social and administrative pharmacy, College of Health Sciences, School of Pharmacy, Addis Ababa University, Addis Ababa, Ethiopia; 2 Department of Preventive medicine , College of Health Sciences, School of Public health, Addis Ababa University, Addis Ababa, Ethiopia; 3 Department of pharmacology and clinical pharmacy, School of pharmacy, College of Health sciences, Addis Ababa University, Addis Ababa, Ethiopia

**Keywords:** Antimicrobial resistance, Self-medication, Sociocultural factors, Healthcare access, Antimicrobial use, Public health, Economic barriers

## Abstract

**Background:**

Antimicrobial resistance (AMR) is a critical global health threat, particularly in low- and middle-income countries (LMICs). In Ethiopia, high infectious disease burdens, unregulated antimicrobial access, and low awareness exacerbate inappropriate use, accelerating AMR. While microbiological aspects of AMR are studied, behavioral and sociocultural factors influencing antimicrobial use remain underexplored. This study investigates these factors in Addis Ababa to inform strategies for mitigating AMR and improving public health outcomes.

**Methods:**

This qualitative study, conducted from April to May 2025 in Addis Ababa, Ethiopia, involved 25 in-depth interviews with patients and the general public across five public health facilities and three community pharmacies. Participants were selected using purposive and convenience sampling techniques. Semi-structured interviews, explored behavioral and sociocultural determinants of antimicrobial use. A codebook was refined iteratively by two coders to ensure consistency. Data were audio-recorded, transcribed, and analyzed using Dedoose software through thematic analysis. Ethical approval was obtained, and confidentiality was maintained throughout. The identified themes were linked with Theoretical domains framework domains to enhance the depth of understanding.

**Results:**

Five key themes emerged: limited knowledge and misconceptions about antimicrobials, with many viewing them as universal cures; prevalent self-medication and non-prescription use, driven by accessibility and trust in pharmacists; varied healthcare-seeking behaviors, influenced by trust and logistical barriers; sociocultural influences, including peer advice and traditional beliefs combining antibiotics with remedies; and structural and economic barriers, such as limited healthcare access and high costs, promoting misuse. These factors, rooted in knowledge gaps, cultural norms, and systemic weaknesses, contribute significantly to inappropriate antimicrobial use and AMR.

**Conclusion:**

The study highlights how misconceptions, self-medication, sociocultural norms, and structural barriers drive inappropriate antimicrobial use in Addis Ababa, exacerbating AMR. Multifaceted interventions are needed, including community education to address knowledge gaps, stricter prescription regulations, improved healthcare access through expanded insurance and clinics, and engagement with cultural leaders to align practices with evidence-based use. These strategies can mitigate AMR while ensuring equitable access to effective treatments, addressing both behavioral and systemic challenges.

**Supplementary Information:**

The online version contains supplementary material available at 10.1186/s40780-025-00503-9.

## Introduction

Escalating global crisis of antimicrobial resistance (AMR) poses a profound threat to public health, demanding urgent and multifaceted interventions to safeguard the efficacy of these vital medications. The World Health Organization (WHO) has unequivocally identified AMR as one of the top ten global health threats facing humanity, emphasizing the potential for a return to a pre-antibiotic era where common infections become untreatable, leading to increased morbidity, mortality, and economic burden [1]. This crisis is particularly acute in low- and middle-income countries (LMICs), where infectious diseases are more prevalent, access to quality healthcare is often limited, and antimicrobial stewardship programs are frequently underdeveloped [2]. Within this global landscape, Ethiopia occupies a particularly vulnerable position, rendering a comprehensive understanding of the factors driving antimicrobial use imperative for effective intervention strategies.

The Ethiopian healthcare system faces a complex interplay of challenges, including a high burden of infectious diseases, limited resources, and significant disparities in access to healthcare services [3]. Infectious diseases, such as pneumonia, diarrhea, and tuberculosis, remain leading causes of morbidity and mortality, particularly among children under five [4]. These conditions often necessitate antimicrobial treatment, creating a substantial demand for these medications. However, the unregulated availability of antimicrobials, coupled with inadequate diagnostic capacity and a lack of awareness among healthcare providers and the general public, contributes to inappropriate and often excessive antimicrobial use [5]. This, in turn, accelerates the development and spread of AMR, threatening the effectiveness of antimicrobial therapies and undermining efforts to control infectious diseases [6].

The prevalence of AMR in Ethiopia is a growing concern, with studies documenting increasing resistance rates to commonly used antimicrobials across a range of bacterial pathogens [7, 8]. This escalating resistance has significant implications for patient outcomes, leading to prolonged hospital stays, increased treatment costs, and a higher risk of mortality [9]. Moreover, the spread of resistant organisms can have far-reaching consequences, affecting not only individual patients but also the broader community [10]. Therefore, a thorough understanding of the factors driving antimicrobial use and the emergence of AMR is crucial for developing effective strategies to mitigate this threat.

While prior studies have explored microbiological aspects of AMR in Ethiopia [7, 8], there remains a critical gap in understanding the behavioral and sociocultural drivers of antimicrobial use, especially in urban settings like Addis Ababa. This study addresses this gap by unpacking specific sociocultural mechanisms such as the role of holy water and spiritual beliefs unique to Ethiopia’s urban context that distinguish antimicrobial use patterns from other LMIC settings. Additionally, it directly compares narratives of patients formally prescribed antimicrobials with those of the general public, a comparison rarely made in existing literature. By providing recent, in-depth qualitative evidence from Addis Ababa, this research offers nuanced, context-specific insights to inform Ethiopia’s national AMR action plan, contributing to tailored strategies for mitigating AMR in urban African settings.

## Methods

### Study setting

This qualitative study was conducted from April to May 2025 across five public health facilities (two health centers and three hospitals) and three community pharmacies in Addis Ababa, Ethiopia. These diverse settings were strategically selected to capture a broad spectrum of perspectives on antimicrobial use and its behavioral and sociocultural determinants. The inclusion of public health facilities representing varying levels of care ensured a range of patient experiences, while community pharmacies served as crucial access points for engaging the general public, including individuals who might not frequently interact with formal healthcare services.

### Study design

This qualitative study used in-depth interviews (IDIs) to explore the behavioral and sociocultural factors influencing antimicrobial use among patients and the general public in Addis Ababa, Ethiopia.

### Study participants

We engaged two key participant groups: patients recently prescribed antimicrobials from public health facilities, and members of the general public. To gain a comprehensive understanding of antimicrobial use behaviors, participants were carefully selected to represent diverse demographics, including variations in age, gender, education level, and socioeconomic status.

### Sample size determination and sampling technique

We enrolled 25 participants in this study. Data collection proceeded until thematic saturation was reached, a process that was monitored iteratively as interviews were conducted. Saturation was formally determined when no new themes or significant concepts emerged from three consecutive interviews, which occurred after the 22nd interview.

We used a multi-pronged sampling approach to ensure a diverse and relevant participant pool. Purposive sampling identified individuals with direct experience in antimicrobial use, ensuring valuable insights into specific behaviors. Concurrently, convenience sampling engaged members of the general public near pharmacies While the use of convenience sampling near pharmacies was pragmatic, we employed maximum variation sampling within this frame by recruiting participants from different socioeconomic backgrounds, ages, and from different facilities to capture a diverse range of perspectives and mitigate selection bias.

### Data collection tool and process

Our data collection employed a semi-structured interview guide designed to explore key themes related to behavioral and socio-cultural determinates of antimicrobial use. To ensure clarity and consistency, the interview guide was initially translated into Amharic, the local language of the study area, and then back-translated into English for verification. Interviews, lasting 30–45 min each, were conducted in Amharic by three trained data collectors. To ensure consistency across interviewers, the data collectors underwent a two-day training workshop. This included a thorough review of the interview guide and refreshment training on the qualitative data collection process. All interviews were audio-recorded and supplemented with detailed field notes to capture additional contexts. To ensure participant comfort and maintain confidentiality, interviews were consistently held in private settings, either within the health facilities or designated community spaces.

### Data analysis

Qualitative data underwent a rigorous, iterative analysis process facilitated by Dedoose software. Initially, a preliminary codebook was developed based on the study objectives and interview guide. To ensure coding consistency and breadth, two independent coders then separately applied this codebook to 20% of the total interview transcripts. During this independent coding phase, additional emergent codes were identified beyond the initial codebook, reflecting the richness of the data. Following this, coding discrepancies were resolved through iterative discussion between the two primary coders until consensus was reached. The refined codebook was then systematically applied to the remaining transcripts. Finally, thematic analysis was employed to identify, analyze, and report patterns (themes) within the data, synthesizing the codes into broader conceptual categories that addressed the study’s qualitative objectives.

To provide depth to the analysis of behavioral and sociocultural factors influencing antimicrobial use, this study employs the Theoretical Domains Framework (TDF), a comprehensive model integrating constructs from multiple behavioral theories into 14 domains, such as knowledge, social influences, and environmental context and resources [11]. The TDF enhances the study’s rigor by systematically mapping qualitative findings to specific behavioral determinants, allowing for a nuanced dissection of individual motivations, social pressures, and systemic barriers that drive inappropriate antimicrobial practices in Addis Ababa [12]. By applying TDF, we move beyond descriptive themes to identify actionable targets for behavior change interventions, offering greater analytical depth and alignment with evidence-based strategies to inform Ethiopia’s national AMR action plan in this urban LMIC context.

### Data quality assurance

This study has employed robust data quality assurance measures throughout the process. Interviews were audio-recorded, meticulously transcribed verbatim, and supplemented with detailed field notes to capture comprehensive insights. Reflexivity was maintained throughout the process by team debriefings to mitigate researcher bias, and member checking was conducted with a subset of three participants to enhance credibility. These participants were purposively selected for their rich and varied perspectives, and they reviewed a thematic outline of our findings.

Their feedback led to minor refinements in the wording of the themes to better reflect their lived experiences.

### Ethical considerations

The study was approved by the research Ethics Review Committee of School of pharmacy, Addis Ababa University. It was conducted in accordance with the Helsinki Declaration. Informed consent was obtained from participants. Confidentiality, neutrality, anonymity, accountability, and academic honesty were maintained throughout the study.

## Results

This section presents the findings from 25 in-depth interviews with patients and members of the general public in Addis Ababa, exploring the behavioral and sociocultural determinants of antimicrobial use. Participants included eleven females and fourteen males, ranging in age from 23 to 50 years. Their educational backgrounds varied from no formal education to university graduates. Ten participants reported chronic diseases, while the others had no known chronic conditions. Their frequency of seeking medical care ranged from rarely/never to often, reflecting diverse engagement with the formal healthcare system (Table 1).

**Table 1:** Demographic characteristics of participants

Category	Subgroup	Number of Participants (*n* = 25)
Gender	Female	11
	Male	14
Age (Years)	18–30 (Young Adults)	10
	31–50 (Middle-Aged)	15
Education Level	Higher Education (Degree+)	7
	Secondary/High School	8
	Primary/No Formal Education	10
Chronic Disease	Hypertension	3
	Diabetes	3
	Asthma	2
	Other (Lupus, RVI)	2
	None	15
*Healthcare-Seeking Frequency	Often	7
	Occasional	6
	Rarely/Never	6
	Not Specified	6

*Healthcare-seeking frequency: ‘Often’ was defined as seeking care more than three times a year; ‘Occasional’ was defined as one to three times a year; ‘Rare’ was defined as less than once a year

**Fig. 1:**
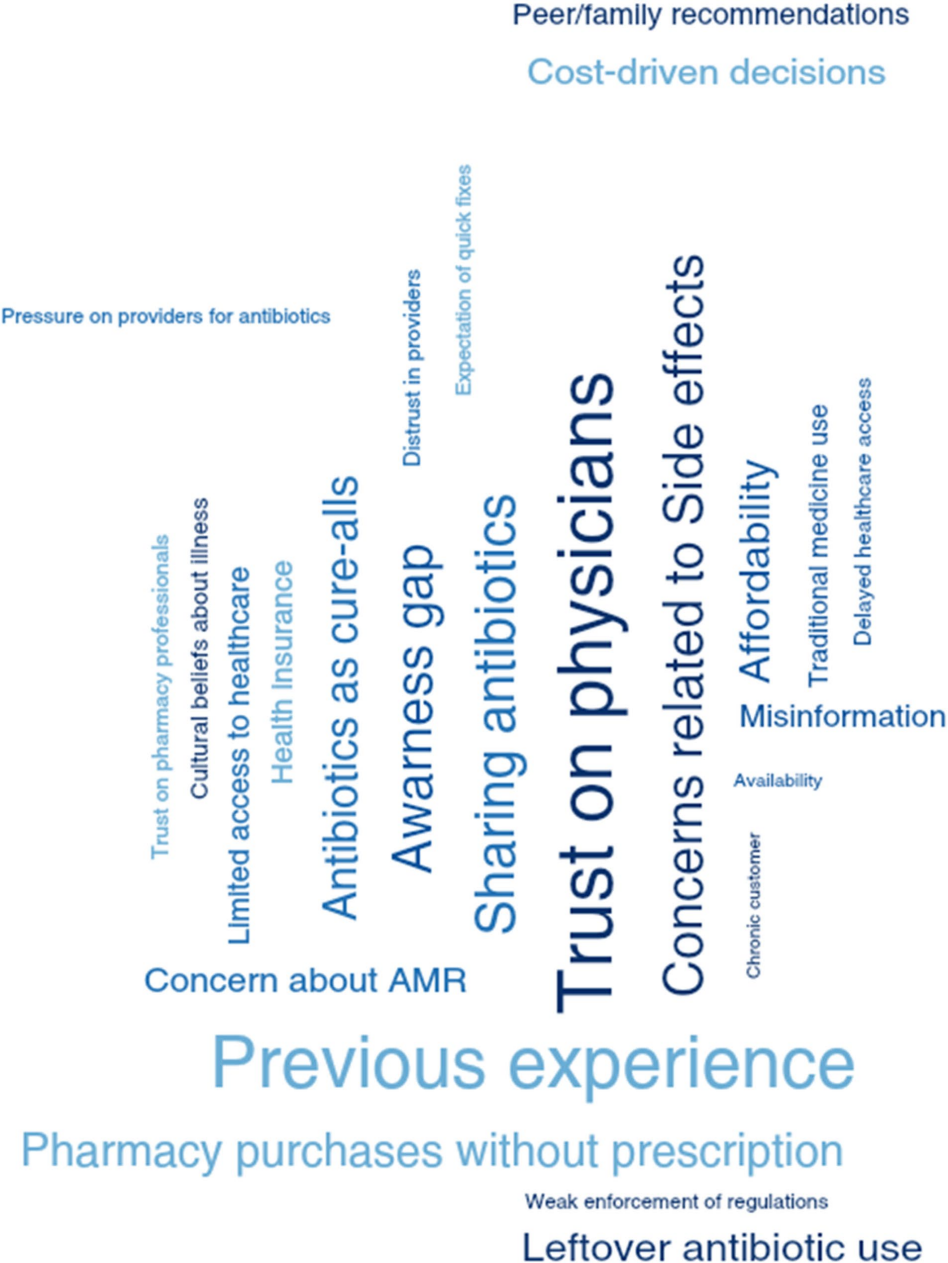
Packed cloud showing frequency occurrence of codes emerged (Dedoose output)

The qualitative in-depth interviews revealed a range of perspectives, behaviors, and barriers related to antimicrobial use. Five key themes emerged from the thematic analysis: Knowledge and Perceptions of Antimicrobials, Self-Medication and Non-Prescription Use, Healthcare-Seeking Behavior, Sociocultural Influences, and Structural and Economic Barriers. Each theme is supported by pertinent quotes from the transcripts (Fig. 1).

### Theme 1: knowledge and perceptions of antimicrobials

While both patient and general public participants demonstrated varying levels of understanding and shared the common misconception that antibiotics are a universal cure for all illnesses, including viral infections, a key contrast emerges in their sources of insight. Patients often cited personal health experiences as a catalyst for their understanding, as illustrated by a 42-year-old male with hypertension who noted, “Some people think antibiotics can cure anything, even colds or stomach issues, which I’ve learned isn’t true” (P2). In contrast, members of the general public more frequently pointed to societal observations, like the 30-year-old female teacher who stated, “Many of my colleagues take them for the flu, which is pointless” (P13). Furthermore, while awareness of antibiotic resistance was linked to educational attainment across both groups, the depth of comprehension differed; a 23-year-old female university student could confidently explain, “Overusing them causes resistance, which means they won’t work in the future” (P6), whereas a 50-year-old male with no formal education showed limited awareness, stating, “I’ve heard they can stop working if you use them too much, but I’m not sure how” (P17). However, both groups were united in their immediate concerns about tangible side effects, such as the 37-year-old male with asthma who noted, “I’ve heard they can mess up your stomach or cause allergies” (P15), suggesting that personal well-being was a common priority that influenced perceptions regardless of participant type.

### Theme 2: self-medication and non-prescription use of antimicrobials

Despite both patients and the general public engaged in self-medication due to the convenience and accessibility of over-the-counter antibiotic purchases, their justifications revealed nuanced differences. A shared trust in pharmacy professionals as accessible alternatives to formal clinics was common, exemplified by a 42-year-old male shopkeeper who admitted, “Yes, many times, it’s easier than going to the clinic, the pharmacist knows what to give” (P10). However, the rationale for other practices often diverged. For members of the public, sharing antibiotics was frequently framed as a communal act of assistance, whereas for patients, particularly those from lower socioeconomic backgrounds, it was more directly a financial necessity, as a 30-year-old female with no formal education shared, “I’ve shared with my kids before because we couldn’t afford more” (P20). Conversely, the practice of using leftover antibiotics was a unifying behavior across both groups, primarily driven by a shared desire to save time and money, a sentiment clearly expressed by a 60-year-old female participant who stated, “Yes, if I have the same sickness again. It saves time and money” (P11).

### Theme 3: healthcare-seeking behavior

Participants’ healthcare-seeking behaviors varied significantly, revealing a clear contrast between those who trusted the formal system and those who circumvented it due to access barriers or distrust. While some patients, particularly those with chronic conditions like the 35-year-old female with lupus, expressed strong confidence in physicians—“Visiting a health care provider is much much better than self-treatment” (P1)—others, often constrained by logistical or financial pressures, delayed care or second-guessed prescriptions. This is exemplified by the 50-year-old male with hypertension who cited distance and lost income, stating, “Going to the clinic is hard it’s far, and I lose a day’s work” (P17), and the 28-year-old female who sought cheaper alternatives: “If the medicine is too expensive, I ask the pharmacist if there’s a cheaper version” (P4). In contrast to these patient-focused barriers, members of the general public were often depicted as a source of external pressure on the healthcare system; a 30-year-old female participant highlighted this dynamic, noting how family influence drives demand: “My mother always insists on getting them for her colds” (P13). Thus, the data shows that while patients grappled with direct structural barriers to appropriate care, the general public could inadvertently perpetuate inappropriate antibiotic demand through informal pressure.

### Theme 4: sociocultural influences

An analysis of sociocultural influences reveals that while both patients and the general public are subject to peer pressure and misinformation, patients managing chronic conditions often demonstrated a more critical awareness of these unreliable sources. The general public was frequently depicted as a direct source of recommendations, as indicated by a 37-year-old male with asthma who reported, “My neighbors often suggest taking antibiotics for small things, like a cough” (P15). In contrast, patients provided specific examples of navigating complex health beliefs, such as a 35-year-old female with diabetes who explained, “People believe in spiritual causes for sickness, so they mix antibiotics with traditional rituals” (P3), highlighting a tension between biomedical and cultural practices. Furthermore, a key difference emerged in the critical evaluation of information; a 25-year-old male with some college education noted the prevalence of poor advice but also his own skepticism, stating, “Mostly from social media and friends, but I know it’s not always reliable” (P14), suggesting that personal health experiences and education can foster a more discerning approach compared to the broader, often uncritical, acceptance within the general public.

### Theme 5: structural and economic barriers

The impact of structural and economic barriers on antibiotic use highlights a critical divergence between the experiences of patients and the general public, particularly in their reliance on the formal healthcare system. Patients, especially those managing chronic diseases, articulated these barriers with specific reference to clinical access and treatment adherence. A 28-year-old female with a chronic disease directly linked facility limitations to informal practices, stating, “Clinics are far, and medicines are sometimes unavailable, so people turn to pharmacies or share drugs” (P16), while a 35-year-old female with lupus highlighted pure affordability: “Most of them are expensive and people do not afford, it is difficult” (P1). For the general public, the same barriers more often manifested as a complete bypass of the formal system from the outset, facilitated by weak enforcement. The consequences of cost also differed; patients framed it as a threat to completing a prescribed course, as a 42-year-old male with vocational training said, “If I didn’t have insurance, I might not finish the course or buy them at all” (P19). In contrast, for the uninsured general public, the financial barrier was absolute, leading to desperate measures like rationing, as a 50-year-old male with hypertension noted about his community, “Many in my area can’t afford antibiotics, so they buy only a few pills” (P17). Thus, while both groups faced similar structural failures, patients were often navigating a system they had tried to access, whereas the public was frequently excluded from it entirely. (Table 2).

**Table 2:** Themes, Codes, and illustrative quotes on antimicrobial use

Theme	Codes	Illustrative Quotes
1. Knowledge & Perceptions	Misconceptions about antibiotics	*“Some people think antibiotics can cure anything*,* even colds or stomach issues*,* which I’ve learned isn’t true.”* (P2, Male, 42)
	Awareness of resistance	*“Overusing them causes resistance*,* which means they won’t work in the future.”* (P6, Female, 23)
	Side effect concerns	*“I’ve heard they can mess up your stomach or cause allergies.”* (P15, Male, 37)
2. Self-Medication & Non-Prescription Use	OTC purchases	*“Yes*,* many times*,* it’s easier than going to the clinic. The pharmacist knows what to give.”* (P10, Male, 42)
	Sharing antibiotics	*“I’ve shared with my kids before because we couldn’t afford more.”* (P20, Female, 30)
	Using leftovers	*“Yes*,* if I have the same sickness again. It saves time and money.”* (P11, Female, 60)
3. Healthcare-Seeking Behavior	Delayed care due to access	*“Going to the clinic is hard—it’s far*,* and I lose a day’s work.”* (P17, Male, 50)
	Trust in providers	*“Visiting a healthcare provider is much better than self-treatment.”* (P1, Female, 35)
	Cost-driven non-adherence	*“If the medicine is too expensive*,* I ask the pharmacist if there’s a cheaper version.”* (P4, Female, 28)
4. Sociocultural Influences	Peer/family pressure	*“My neighbors often suggest taking antibiotics for small things*,* like a cough.”* (P15, Male, 37)
	Traditional beliefs	*“People believe in spiritual causes for sickness*,* so they mix antibiotics with traditional rituals.”* (P3, Female, 35)
	Misinformation	*“Mostly from social media and friends*,* but I know it’s not always reliable.”* (P14, Male, 25)
5. Structural & Economic Barriers	Limited healthcare access	*“Clinics are far*,* and medicines are sometimes unavailable*,* so people turn to pharmacies or share drugs.”* (P16, Female, 28)
	Affordability issues	*“Most of them are expensive*,* and people do not afford. It is difficult.”* (P1, Female, 35)
	Weak regulation enforcement	*“Pharmacies shouldn’t sell antibiotics without prescriptions—it’s too easy to get them.”* (P19, Male, 4

### Linkage of the themes with theoretical domains framework

This study’s findings, analyzed through the Theoretical Domains Framework (TDF), reveal a complex interplay of behavioral determinants driving inappropriate antimicrobial use in Addis Ababa, Ethiopia. The first theme, Knowledge and Perceptions of Antimicrobials, aligns with TDF domains such as knowledge, where misconceptions about antibiotics as universal cures (e.g., P2) reflect limited understanding, and beliefs about consequences, including awareness of AMR risks (e.g., P6) and side effects (e.g., P15), which vary by education level and influence adherence. The second theme, Self-Medication and Non-Prescription Use, maps to environmental context and resources, evidenced by easy OTC access (e.g., P10) and economic pressures (e.g., P20), alongside social influences from trusted pharmacists and intentions driven by convenience (e.g., P11), highlighting poor behavioral regulation in practices like sharing or using leftovers. The third theme, Healthcare-Seeking Behavior, corresponds to environmental context and resources, with barriers like clinic distance (e.g., P17) and costs (e.g., P4) reducing motivation and goals for formal care, while social/professional role and identity is evident in varying trust in providers (e.g., P1) versus family-driven demands (e.g., P13).

Extending the TDF analysis, the fourth theme, Sociocultural Influences, integrates with social influences, where peer recommendations (e.g., P15) and misinformation from social media (e.g., P14) shape behaviors, and beliefs about consequences tied to cultural attributions of illness (e.g., P3), such as spiritual causes leading to blended remedies, reinforcing community norms in social/professional roles. Finally, the fifth theme, Structural and Economic Barriers, primarily aligns with environmental context and resources, including limited healthcare access (e.g., P16) and affordability issues (e.g., P1), which undermine motivation and goals for complete courses (e.g., P17), compounded by weak behavioral regulation due to lax enforcement (e.g., P19). Collectively, these TDF mappings underscore how individual cognitive factors intersect with social and environmental determinants, providing a granular foundation for targeted interventions to mitigate AMR in urban Ethiopian settings.

## Discussion

This study applied the Theoretical Domains Framework (TDF) to explore the behavioral and sociocultural factors driving antimicrobial use in an urban African context, Addis Ababa, Ethiopia. The study’s novelty lies in its unique application of the TDF to this specific setting, which provides a novel, theory-informed analysis unlike previous descriptive reports in the region. Our deeper use of the TDF allowed us to move beyond a simple description of themes and instead systematically map the identified drivers to specific theoretical domains. This approach provides a robust and clear theoretical foundation for designing targeted, evidence-based public health interventions to combat antimicrobial resistance (AMR).

Our findings, derived from rich qualitative data, reveal several unique urban factors, including the ease of non-prescription antibiotic access from numerous pharmacies, the influence of urban peer networks on medication sharing, and culturally specific beliefs, such as the concurrent use of holy water and antibiotics.

The study revealed varying levels of understanding about antimicrobials, with misconceptions such as antibiotics being a universal cure for all illnesses, including viral infections, being prevalent. This aligns with findings from a systematic review reported that only 51.53% of the population has good knowledge of AMR, indicating widespread gaps in awareness [13]. Similarly, another study from Uganda stated that cultural beliefs in antibiotics as a cure-all encourage misuse [14]. The awareness of antibiotic resistance among some participants, particularly those with higher education, corroborates the report of Italian study, which found that higher educational attainment correlates with better understanding and responsible antibiotic use [15]. However, the limited awareness among others, especially those with lower education levels, highlights a critical need for targeted educational interventions, as suggested by a study from Nigeria [16]. Concerns about side effects, such as stomach issues or allergies, also shaped perceptions, which may deter some from completing antibiotic courses, further contributing to resistance.

Self-medication and non-prescription use of antibiotics were prevalent, driven by convenience, trust in pharmacists, and economic constraints. Participants frequently purchased antibiotics over the counter (OTC) or used leftovers, consistent with another study from Addis Ababa, Ethiopia evaluating self-medication practices [17] and Eastern Ethiopia, which reported that 45.6% of individuals access antibiotics inappropriately due to easy availability and economic pressures [18]. The high prevalence of self-medication across Africa (12.1% to 93.9%), as noted by a comparative study in Africa [19], underscores the regional challenge of unregulated antibiotic dispensing. Sharing antibiotics with family or friends, often viewed as a cost-saving measure, further exacerbates misuse, aligning with sociocultural practices reported by a study from Nigerian southwestern state [20]. These findings suggest that trust in pharmacists, while beneficial for accessibility, may undermine efforts to curb AMR unless accompanied by stricter regulations and public education on the risks of non-prescribed use.

Healthcare-seeking behaviors were influenced by logistical challenges, trust in providers, and cost concerns, leading to delayed care or reliance on pharmacies. Participants’ reluctance to visit clinics due to distance or work loss mirrors findings of Eastern Ethiopia [18], which highlighted healthcare access barriers in Ethiopia. Trust in healthcare providers, as expressed by some participants, aligns with a study from South Africa [21], who emphasized the role of patient-provider dynamics in responsible antibiotic use. However, pressure on providers for antibiotics, often driven by family expectations, reflects a cultural tendency noted by a UK study [22]. This pressure, combined with patients second-guessing prescriptions due to cost or perceived inefficacy, suggests a need for improved communication strategies to align patient expectations with evidence-based prescribing practices.

Sociocultural factors, including peer recommendations and cultural beliefs about illness, significantly shaped antibiotic use. The influence of social networks, as reported by participants, aligns with the report of UK’s study, which identified social norms as a key driver of antibiotic prescribing and consumption [23]. Cultural beliefs linking illnesses to non-biomedical causes, such as the “bird,” led to the integration of antibiotics with traditional remedies, a practice also noted by a Ugandan study [14]. Misinformation from social media and community sources, as highlighted by participants, underscores the role of media in shaping public perceptions, as discussed by Wojcik et al. [24]. These findings indicate that interventions must address not only individual knowledge but also community-level beliefs and misinformation channels to promote responsible antibiotic use.

Structural and economic barriers, such as limited healthcare access and high costs, were significant drivers of inappropriate antibiotic use. Participants’ reliance on pharmacies due to distant or unavailable clinics aligns with Eastern Ethiopian study [18], who noted that healthcare system limitations in Ethiopia contribute to non-prescribed antibiotic use. The economic burden of antibiotics, leading to incomplete courses or sharing, corroborates the findings of studies, which highlighted socioeconomic disparities in healthcare access in settings like India [25] and South Africa [21]. Weak regulatory enforcement, allowing OTC antibiotic sales, is a critical issue also reported by studies across Africa [14, 20]. While health insurance mitigated some barriers, its absence exacerbated vulnerabilities, as noted by participants. These findings contrast with arguments that stricter regulations alone may not address deep-seated cultural practices or systemic healthcare inadequacies, as suggested by Belachew et al. [17], emphasizing the need for a multifaceted approach.

The study reveals a critical conflict between immediate individual health demands and long-term public health objectives, with individuals favoring accessibility and affordability over responsible antibiotic use, consistent with prior research [26]. In contrast to global studies focusing on patient-provider interactions [24], this research emphasizes pharmacies as accessible yet unregulated antibiotic sources, particularly in resource-limited settings. The blending of traditional remedies with antibiotics, specific to certain cultural contexts, introduces complexities not fully explored in broader African studies [14, 20]. The findings align with existing literature on antimicrobial resistance (AMR) drivers in low- and middle-income countries (LMICs), especially in Ethiopia and Africa [14, 17, 20, 21], highlighting persistent knowledge gaps, widespread self-medication driven by convenience and economic barriers, and the influence of social and cultural norms. Crucially, the study underscores how healthcare system weaknesses and economic disparities intensify these behavioral and sociocultural challenges, creating a complex environment that undermines appropriate antimicrobial use.

### Limitation

This study is subject to important limitations. The findings are context-specific to an urban setting and may not be transferable to rural populations where healthcare access and sociocultural dynamics differ substantially. Although maximum variation sampling was used, the recruitment strategy near pharmacies likely excluded the most marginalized individuals without any access to these services. The qualitative design relies on self-reported data, which is vulnerable to social desirability and recall bias, particularly regarding sensitive behaviors like self-medication. Additionally, while the inter-rater agreement between coders was achieved through discussion and consensus, no statistical measures of inter-rater reliability, such as Cohen’s kappa, were calculated. Finally, while the sample size was sufficient for thematic saturation, it limits the generalizability of the findings across Ethiopia’s diverse populations.

## Conclusion

The findings of this qualitative study underscore the profound influence of knowledge gaps, prevalent self-medication practices, diverse healthcare-seeking behaviors, powerful sociocultural norms, and significant structural and economic barriers on antimicrobial use among the general public. Misconceptions about antibiotics as universal cures, coupled with the ease of non-prescription access and the economic pressures on individuals, create a fertile ground for inappropriate use. Sociocultural influences, including peer advice and traditional beliefs, further complicate adherence to responsible practices, while systemic issues like limited healthcare access and weak regulatory enforcement exacerbate the problem. To mitigate AMR, a multifaceted strategy is essential, combining targeted public education, stricter enforcement of prescription policies, improved healthcare accessibility, and the engagement of community leaders to align cultural practices with rational antibiotic use.

## Supplementary Information

Supplementary Material 1.

## Data Availability

Data is provided within the manuscript.
