# Supplementary material for: Unraveling behavioral and sociocultural factors that shape antimicrobial use among patients and general public, Addis Ababa, Ethiopia, a qualitative study
Source: J Pharm Health Care Sci. 2025 Oct 29;11:97. doi: 10.1186/s40780-025-00503-9 (PMC12574236; doi:10.1186/s40780-025-00503-9)
Supplement: Supplementary file 1 — Supplementary Material 1. [file 40780_2025_503_MOESM1_ESM.docx]

**Interview guide _ English version**

**Title: Unraveling behavioral and sociocultural factors that shape antimicrobial use among patients and general public, Addis Ababa, Ethiopia, a qualitative study**

**Dear Sir/Madam,**

**Demographic information:**

- Gender_________
- Age (in years)________
- Level of education________
- Do you have any chronic health conditions? If so, please specify.__________
- How often do you seek medical care for infections or illnesses?___________

**[Start the audio recording]**

1. What are your Beliefs about Antimicrobials use?
   - What is your perception of antibiotics for treating infections?
   - In your experience, how effective have antimicrobials been for your health?
   - What are the risks you associate with antimicrobial use?
   - What misconceptions do you think people have about antibiotics?
   - What do you think happens if antibiotics are not taken as prescribed or are overused?
2. How would you describe your level of health literacy?
   - How well do you understand the information about antimicrobials provided by healthcare professionals?
   - Do you feel confident discussing your treatment options?
   - How do you seek information about antibiotics when you have questions?
3. How does your past experience with antimicrobials affect your use practice?

- Can you recall a past experience where you or someone close to you was prescribed antibiotics? How did it influence your opinion about their use?
- Have you ever had an experience where antibiotics didn’t work as expected? What did you think or feel at the time?
- How did previous experiences with antibiotics shape your expectations for future treatments?

1. How would you describe the external factors that influence antimicrobial use?
   - What factors influence your decision to take antibiotics when prescribed?
   - Do you ever seek antibiotics from sources outside the formal healthcare system (e.g., pharmacies, friends, or family)? Why?
   - What role does convenience (e.g., pharmacy access, short-term relief) play in your decision to use antibiotics?
   - Have you ever second-guessed a healthcare provider’s decision to prescribe antibiotics? Why?
2. How does the cost of antimicrobials impact your antimicrobial use practices?
   - How do the prices of antibiotics influence your decision to use them?
   - Have you ever avoided getting a prescription due to unavailability?
   - How does your insurance coverage impact your decision to seek antibiotic treatment?
3. How do social Norms influence your antimicrobial use?
   - How do friends or family influence your decisions regarding antibiotic use?
   - Is there pressure from your social circle to use antibiotics for certain illnesses?
   - Do you notice differences in antibiotic use among different social groups you belong to?
   - Have you faced difficulties obtaining prescriptions for antibiotics?
4. What is your perception towards drug Sharing and Use of Leftover antimicrobial use?
   - Have you ever shared antibiotics with friends or family? Why or why not?
   - What are your views on sharing medications with others?
   - Do you ever use leftover antibiotics from previous prescriptions? Why?
5. What are the barriers to proper use of antimicrobials?
   - What barriers do you face in using antimicrobials appropriately?
   - Are there financial, educational, or accessibility barriers?
   - How do these barriers impact your health decisions?

**Closing**

1. Is there anything important that we haven't discussed, and you feel should be included in our study?

Thank you for sharing your experiences and insights. Your input is invaluable to our research.

**[Stop the audio recording]**

**Table 1: Demographic Characteristics of Participants**

| Category | Subgroup | Number of Participants (n=25) |
| --- | --- | --- |
| Gender | Female | 13 |
|  | Male | 12 |
| Age (Years) | 18–30 (Young Adults) | 10 |
|  | 31–50 (Middle-Aged) | 15 |
| Education Level | Higher Education (Degree+) | 7 |
|  | Secondary/High School | 8 |
|  | Primary/No Formal Education | 10 |
| Chronic Disease | Hypertension | 3 |
|  | Diabetes | 3 |
|  | Asthma | 2 |
|  | Other (Lupus, RVI) | 2 |
|  | None | 15 |
| Healthcare-Seeking Frequency | Often (Monthly–Quarterly) | 7 |
|  | Occasional | 6 |
|  | Rarely/Never | 6 |
|  | Not Specified | 6 |

**Table 2: Themes, Codes, and Illustrative Quotes on Antimicrobial Use**

| Theme | Codes | Illustrative Quotes |
| --- | --- | --- |
| 1. Knowledge & Perceptions | Misconceptions about antibiotics | *"Some people think antibiotics can cure anything, even colds or stomach issues, which I’ve learned isn’t true."* (P2, Male, 42) |
|  | Awareness of resistance | *"Overusing them causes resistance, which means they won’t work in the future."* (P6, Female, 23) |
|  | Side effect concerns | *"I’ve heard they can mess up your stomach or cause allergies."* (P15, Male, 37) |
| 2. Self-Medication & Non-Prescription Use | OTC purchases | *"Yes, many times, it’s easier than going to the clinic. The pharmacist knows what to give."* (P10, Male, 42) |
|  | Sharing antibiotics | *"I’ve shared with my kids before because we couldn’t afford more."* (P20, Female, 30) |
|  | Using leftovers | *"Yes, if I have the same sickness again. It saves time and money."* (P11, Female, 60) |
| 3. Healthcare-Seeking Behavior | Delayed care due to access | *"Going to the clinic is hard—it’s far, and I lose a day’s work."* (P17, Male, 50) |
|  | Trust in providers | *"Visiting a healthcare provider is much better than self-treatment."* (P1, Female, 35) |
|  | Cost-driven non-adherence | *"If the medicine is too expensive, I ask the pharmacist if there’s a cheaper version."* (P4, Female, 28) |
| 4. Sociocultural Influences | Peer/family pressure | *"My neighbors often suggest taking antibiotics for small things, like a cough."* (P15, Male, 37) |
|  | Traditional beliefs | *"People believe in spiritual causes for sickness, so they mix antibiotics with traditional rituals."* (P3, Female, 35) |
|  | Misinformation | *"Mostly from social media and friends, but I know it’s not always reliable."* (P14, Male, 25) |
| 5. Structural & Economic Barriers | Limited healthcare access | *"Clinics are far, and medicines are sometimes unavailable, so people turn to pharmacies or share drugs."* (P16, Female, 28) |
|  | Affordability issues | *"Most of them are expensive, and people do not afford. It is difficult."* (P1, Female, 35) |
|  | Weak regulation enforcement | *"Pharmacies shouldn’t sell antibiotics without prescriptions—it’s too easy to get them."* (P19, Male, 4 |
